# Supplementary material for: Variability of cholesterol accessibility in human red blood cells measured using a bacterial cholesterol-binding toxin
Source: eLife. 2017 Feb 8;6:e23355. doi: 10.7554/eLife.23355 (PMC5323040; doi:10.7554/eLife.23355)
Supplement: Table 5—source data 1. — DOI: http://dx.doi.org/10.7554/eLife.23355.016 [file elife-23355-table5-data1.docx]

| **Table 5 – source data 1.** Correlation between fatty acids (FA) and fALOD4 binding. | | | | |
| --- | --- | --- | --- | --- |
| **FA (ug FA/ug protein)** | **Spearman’s rho** | **P-value (Spearman)** | **Partial r** | **P-value (linear regression)** |
| C16: 0 (Palmitic) | -0.06 | 0.59 | -0.08 | 0.50 |
| C18:0 (Stearic) | 0.01 | 0.95 | -0.04 | 0.75 |
| C18:1n9c (Oleic) | -0.10 | 0.37 | -0.09 | 0.42 |
| C18:2n6c (Linoleic) | 0.06 | 0.59 | -0.02 | 0.85 |
| C20:3n6 (cis-8,11,14-Eicosatrienoic) | -0.05 | 0.65 | -0.04 | 0.72 |
| C20:4n6 (Arachidonic) | 0.10 | 0.39 | 0.05 | 0.63 |
| C20:5n3 (cis-5,8,11,14,17-Eicosapentaenoic) | -0.01 | 0.92 | -0.04 | 0.70 |
| C24:1n9 (Nervonoic) | -0.06 | 0.60 | -0.03 | 0.77 |
| C22:4 Docosatetranoic | 0.11 | 0.33 | 0.07 | 0.52 |
| C22:5n3 Docosapentanoic | 0.06 | 0.56 | 0.00 | 0.98 |
| C22:6n3 (cis-4,7,10,13,16,19-Docosahexaenoic) | 0.15 | 0.18 | 0.01 | 0.93 |
| Fatty acids were measured on lipids extracted from the RBCs of Biobank participants as reviewed in the Methods. The demographics and clinical characteristics of the Biobank participants is shown in Table 5. Partial correlation (r) was calculated using linear regression of fALOD4 binding on the fatty acid, adjusting for age, sex, and ethnicity. | | | | |
